# HCV-related liver and lymphoproliferative diseases: association with polymorphisms of IL28B and TLR2

**DOI:** 10.18632/oncotarget.9303

**Published:** 2016-05-11

**Authors:** Valli De Re, Mariangela De Zorzi, Laura Caggiari, Gianfranco Lauletta, Maria Lina Tornesello, Elisa Fognani, Marta Miorin, Vito Racanelli, Luca Quartuccio, Laura Gragnani, Sabino Russi, Fabio Pavone, Michela Ghersetti, Elena Garlatti Costa, Pietro Casarin, Riccardo Bomben, Cesare Mazzaro, Giancarlo Basaglia, Massimiliano Berretta, Emanuela Vaccher, Francesco Izzo, Franco Maria Buonaguro, Salvatore De Vita, Anna Linda Zignego, Paolo De Paoli, Riccardo Dolcetti

**Affiliations:** ^1^ Bio-Proteomics Facility/ Cancer Bioimmunotherapy, Department of Translational Research, Centro di Riferimento Oncologico (CRO), National Cancer Institute, Aviano, Italy; ^2^ Liver Unit, Division of Internal Medicine and Clinical Oncology, Department of Biomedical Sciences and Human Oncology, University of Bari Medical School, Bari, Italy; ^3^ Molecular Biology and Viral Oncology Unit, Istituto Nazionale Tumori “Fondazione G. Pascale” - IRCCS, Napoli, Italy; ^4^ Interdepartmental Center for Systemic Manifestations of Hepatitis Virus MASVE, Department of Experimental and Clinical Medicine, University of Florence, Florence, Italy; ^5^ Cytogenetics and Molecular Biology Unit, Santa Maria degli Angeli Hospital Pordenone, Pordenone, Italy; ^6^ Immunology Section, Department of Biomedical Sciences and Human Oncology, University of Bari Medical School, Bari, Italy; ^7^ Clinic of Rheumatology, Department of Medical and Biological Sciences, University Hospital “Santa Maria della Misericordia”, Udine, Italy; ^8^ Internal Medicine-Liver Unit, Santa Maria degli Angeli Hospital Pordenone, Pordenone, Italy; ^9^ Clinical and Experimental Onco-Hematology Unit, Centro di Riferimento Oncologico (CRO), National Cancer Institute, Aviano, Italy; ^10^ Microbiology-Immunology and Virology Unit, Centro di Riferimento Oncologico (CRO), National Cancer Institute, Aviano, Italy; ^11^ Medical Oncology, Centro di Riferimento Oncologico (CRO), National Cancer Institute, Aviano, Italy; ^12^ Hepatobiliary Unit, Istituto Nazionale Tumori “Fondazione G. Pascale” - IRCCS, Napoli, Italy; ^13^ Scientific Directorate, Centro di Riferimento Oncologico (CRO), National Cancer Institute, Aviano, Italy; ^14^ Cancer Bio-Immunotherapy, Department of Translational Research, Centro di Riferimento Oncologico (CRO), National Cancer Institute, Aviano, Italy; ^15^ University of Queensland Diamantina Institute, Translational Research Institute, Brisbane, Queensland, Australia

**Keywords:** HCV, TLR2, IL28B, HCC, NHL, Immunology and Microbiology Section, Immune response, Immunity

## Abstract

To explore the relationship between innate immunity and hepatitis C Virus (HCV) in determining the risk of cirrhosis (CIR), hepatocellular carcinoma (HCC), mixed cryoglobulinemia syndrome (MCS) and non-Hodgkin lymphoma (NHL), we investigated the impact of the toll-like receptor-2 (TLR2) and interleukin-28B (IL28B) genetic variants. TLR2 −174 del variant was associated with TLR2 expression and with specific downstream molecules that drive the expression of different interleukins; rs12979860 Il28B was important in response to interferon-treatment and in spontaneous clearance of HCV. The risk for liver and lymphoproliferative diseases in HCV progression was clarified by stratifying 862 HCV-positive patients into groups based on liver (CIR, HCC) and lymphoproliferative HCV-related diseases (MCS, NHL) and compared with chronic HCV (CHC) infection. Analysis of TLR2-IL28B haplotypes showed an association of wild type haplotype with the lymphoproliferative diseases (OR 1.77, *p* = 0.029) and a slight increase in HCV viral load (HR 1.38, p = 0.054). Wild type haplotype (TLR2 ins/ins- IL28B C/C) was also found associated with older age in patients with an hepatic diseases (in CIR and in HCC *p* = 0.038 and *p* = 0.020, respectively) supporting an effect of innate immunity in the liver disease progression. TLR2 and IL28B polymorphisms in combination showed a role in the control of HCV viral load and different HCV disease progression.

## INTRODUCTION

Chronic HCV-infection (CHC) may induce cirrhosis and hepatocellular carcinoma (HCC), but also B-cell dysregulations such as autoimmune type II mixed cryoglobulinemia syndrome (MCS) and B-cell non-Hodgkin lymphoma (NHL) [1–3]. The mechanisms whereby HCV establishes hepatic or lymphoproliferative diseases are still poorly understood.

Innate immune response relies on recognition of pathogen-associated molecular patterns (PAMP) through pattern recognition receptors (PRRs) that include the toll-like receptors (TLRs). TLR2, TLR3, TLR7 and TLR9 have a crucial role in host defense against HCV infection and HCV liver diseases [4–7]. Like other TLRs (TLR1, TLR4, TLR5, TLR6 and TLR10), TLR2 is predominantly expressed on the cell surface to sense extracellular PAMPs. In contrast, TLR3, TLR7, TLR8 and TLR9 are located in intracellular compartments and recognize viral nucleotides. TLR2 has been associated with hepatocarcinogenesis and is known to be triggered by the core-protein of the HCV nucleocapsid [8–12]. Intriguingly, specific TLR polymorphisms, mainly the TLR2 −196 to −174 ins/del, influence the extent of TLR2 expression and have been associated with more advanced liver diseases [13, 14]. TLRs signal through specific downstream molecules that finally activate transcription factors driving the expression of different cytokines including interferons [15, 16].

Inappropriate production of interferons has been associated to various HCV-related processes that may be the prelude to malignant complications [17, 18]. Interferon-lamba (IFNλ) is a family of four genes: IFNλ1 (IL29), IFNλ2 (Il28A), IFNλ3 (IL28B) and IFNλ4 (a frameshift variant of IL28B) [19]. They target the IL28Rα and IL10Rb receptors resulting in the activation of Janus kinase/signal transducers and activators of transcription (JAK/STAT) signaling with expression of interferon-stimulated genes (ISGs) and induction of an antiviral state. However, unlike type I IFN receptor, which was expressed on virtually all cell types, IL28Rα was only expressed on specific tissues such as epithelia [20]. A single nucleotide polymorphism (SNP) in the promoter region of the IL28B gene, the rs12979860, was originally associated with spontaneous and treatment-induced clearance of genotype 1 HCV infection [2, 17, 21–26].

We hypothesized that, if TLR2 and IL28B were functionally interconnected in HCV diseases-specific networks, both TLR2 and IL28B polymorphisms could have a critical role in the pathogenesis of HCV-related disorders.

To test this hypothesis, we investigated TLR2 −196 to −174 ins/del and rs12979860 IL28B polymorphisms in HCV-related liver and lymphoproliferative diseases in an Italian population.

## RESULTS

### Patients characteristics and group distribution

Patient characteristics and group distribution were shown in Table 1. Gender and HCV genotypes were found differentially distributed among HCV-related disease groups. Male gender was preferentially found associated with liver diseases (CIR and HCC; 67 to 71%), while female gender was more frequent in patients with lymphoproliferative disorders (MCS and NHL; 63 and 61%). HCV genotype 1 was the most frequent genotype found in our population (53 to 82%). No association between HCV genotypes and HCV viral load was observed.

**Table 1:** Main clinical and laboratory findings of HCV chronically infected patients according to liver-related damage and extrahepatic conditions

	HCV neaative	HCV-positive patients				
	Blood donors		Liver diseases		lymphoproliferative diseases	
		Chronic HCV infection	Cirrhosis	Hepatocellular carcinoma	Cryoglobulinemia	Non Hodgkin lymphoma
	No. 77	No. 230	No. 123	No. 175	No. 205	No. 129
**Age**						
Mean (± SD)	42 (± 10)	55 (± 13)	63 (± 12)	69 (± 10)	62 (± 12)	69 (± 13)
**Gender**						
Male	60 (78%)	131(57%)	87(71%)	117(67%)	75 (37%)	50 (39%)
**HCV genotypes**						
1	-	112(60%)	64 (52%)	129(78%)	78 (62%)	45 (82%)
2	-	46 (24%)	24 (20%)	19 (12%)	33 (26%)	7(13%)
3–4	-	30 (16%)	21 (17%)	16(10%)	14 (12%)	2(5%)
Nd	-	42	14	11	80	75
Negative	77(100%)	-	-	-	-	-
Median HCV load (lU/ml) (min-max*1000)	-	1,522,000 (1-18,810)	782,000 (4,6-13,6)	2,766,000 (14-4,075)	1,730,000 (5-22,669)	1,100,000 (1-12,300)

Nd: HCV genotype not determined

### Genotype frequencies of TLR2 ins/del polymorphism in chronic HCV infection, HCV-related liver and lymphoproliferative diseases

**Figure 1:**
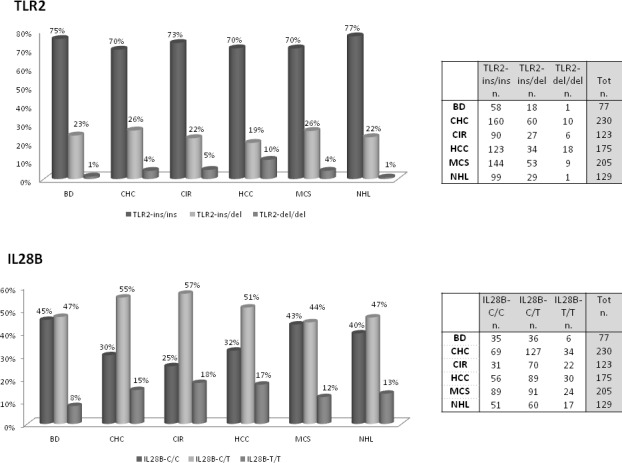
Distribution of TLR2 and IL28B genotypes among HCV-positive population groups TLR2 and IL28B genotypes frequencies and relative number of cases in patients with chronic HCV infection (CHC), with HCV-related cirrhosis (CIR), with HCV-related hepatocellular carcinoma (HCC), with HCV-related mixed cryoglobulinemic syndrome (MCS), with HCV-related non Hodgkin lymphoma (NHL) and in blood donors (BD).

**Figure 2:**
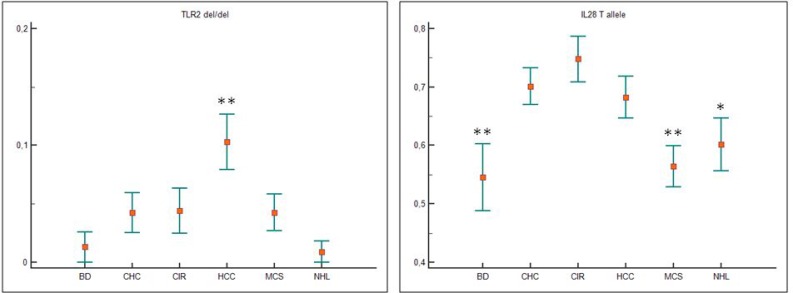
One-way Anova test for TLR2 and IL28B variant alleles and for TLR2 and IL28B genotypes Data were represented as mean with Standard Error of measurement (±SEM). Only statistically significant comparisons were illustrated. The del/del TLR2 genotype was significantly higher in patients with HCV-related hepatocellular carcinoma (HCC) compared to all the other groups (*p* = 0.004) (**). Patients with HCV-related liver damage showed a higher IL28B T allele mean value than blood donors (BD) (*p* = 0.001, CHC reference group) and patients with lymphoproliferative diseases (MSC and NHL, *p* = 0.012, CHC reference group).

Figure 1 shows the genotype frequencies of TLR2 ins/del (−196 to −174) polymorphism in patients with HCV, HCV-related diseases and blood donors (BD). Distribution of BD TLR2 ins/del genotype frequencies in our case series was similar to that reported in the literature [13]. Carriers of wild-type TLR2 (ins/ins) genotype represented 71.6 % of all HCV-positive patients, with a frequency of 70% to 77% in the different HCV-related diseases, and 75% in BD (Figure 1). Data indicated a slight association between TLR2-del allelic variant with HCV infection, but the difference was not significant when polymorphism frequencies of all HCV-positive patients were compared to those of BD (dominant TLR2 ins/ins *vs* TLR2 ins/del + TLR2 del/del, OR = 1.20, Table 2). However, patients carrying TLR2 del/del genotype showed an increased risk for HCC compared with all other groups of patients and BDs by using pair wise comparison test (one-way analysis of variance HCC *vs* NHL *p* = 0.004; Figure 2). In particular, a positive trend between TLR2 del/del genotype and an increasing risk for HCC (i.e CHC: 4.3%; CIR: 4.9%; HCC: 10.3%) was found; by contrast, the TLR2 del/del prevalence in NHL patients (0.9%) was similar to that found in BDs (1.3%). Thus, findings indicated that TLR2 del/del genotype condition correlate with primary liver cancer rather than HCV-positive malignant lymphoproliferation. No additional significant differences was observed for TLR2 ins/del genotype or allele frequencies.

### Genotype frequencies of rs12979860 IL28B polymorphism in chronic HCV infection, HCV-related liver and lymphoproliferative diseases

The results of IL28B genotyping in patients with HCV-related diseases and BDs were shown in Figure 1. Distribution of rs12979860 genotype frequencies found in our series was similar to those reported in the literature [17, 21, 22]. Results of previous studies where IL28B C/C genotype enhanced the spontaneous resolution of HCV infection indirectly support a possible role for the IL28B C allele in the control of HCV infection. In keeping with these observations, we found a negative trend for CC genotype frequencies, balanced by an increased trend in TT genotype, in HCV-positive patients compared with BDs (IL28B C/C dominant polymorphism *vs* IL28B C/T+ IL28B T/T: OR 0.46 *p* = 0.025, Table 2). One-way test confirmed the significant positive trend for T allele in patients with chronic HCV-infection, which was more evident in patients with liver damage instead of a lymphoproliferative disorder (Figure 2). Thus, the analysis indicated a T allele gradient able to discriminate a chronic status more prone to evolve into liver injury from a condition more likely developing a lymphoproliferative dysfunction (*p* = 0.001, Figure 2). The frequency of IL28B T allele in CHC, CIR, HCC was 43.7%, in MCS, NHL was 35.1% in BDs was 27.2%.

**Table 2:** Genotype associations with HCV infection

	Dominant		Recessive		Additive 1		Additive 2	
Gene polymorphism	*P*-value	OR (95% CI)	*P*-value	OR (95% CI)	*P*-value	OR (95% CI)	*P*-value	OR (95% CI)
TLR2 (ins→del)	0.521	1.20 (0.69-2.06)	0.168	4.09 (0.47 −1.41)	0.325	0.76 (0.43-1.32)	0.167	4.118 (0.55-30.61)
IL28B rs12979860 (C→T)	0.025	0.46 (0.28-0.76)	0.260	1.99 (0.60-6.58)	0.082	1.84 (0.92-3.66)	0.075	3.05 (0.89-10.41)

HCV-positive patients (n= 862) were compared with respect to the reference category of blood donors (n=77). OR, odds ratio; CI, confidential interval. WT: wild type is the most frequent polymorphism found in the general population. For the TLR2 SNP indicated like *ins* and *del* variant; a single band at 286 bp was judged as WT, while heterozygous type revealed two bands of 286 bp and 264 bp (*del* variant). Genotypes of each polymorphism were assessed according to dominant (1 wild-type homozygote; 0 heterozygote and variant homozygote), recessive (0 wild-type homozygote and heterozygote; 1 variant homozygote) and additive genetic models. Additive models comprised additive 1 (1 heterozygotes versus 0 wild-type homozygotes) and additive 2 (1 variant homozygotes versus 0 wild-type homozygotes) models. Dominant indicated homzygous WT alleles, which was for the TLR2 the ins/ins genotype and for the IL28B the C/C genotype.

### TLR2-IL28B haplotypes in chronic HCV infection, HCV-related liver and lymphoproliferative diseases

No linkage disequilibrium between TLR2 and IL28B polymorphisms was found as the test for correlation resulted not significant (862 data pairs tested; coefficient of correlation 0.03). The TLR2-IL28B polymorphisms formed several haplotypes, whose frequency was reported on Table 3. The distribution analysis of haplotypes confirmed that specific haplotypes discriminated between HCV-positive patients more likely evolving towards liver damage and those instead evolving towards a lymphoproliferative disorder. Indeed, a significant lack of the TLR2-ins/ins IL28B-C/C haplotype was not only associated with a chronic HCV status (*p* = 0.007, OR 2.94, Table 4) but closely correlated with a liver damage, rather than a lymphoproliferative-disease progression (*p* = ns, OR 1.05; *p* = 0.029, OR 1.77, respectively, Table 4).

**Table 3:** Frequencies of TLR2 /IL-28B haplotypes based on HCV-related diseases and blood donors (BD)

TLR2	IL28B	frequency					
		BD N=77	CHC N=230	CIR N=123	HCC N=175	MCS N=205	NHL N=129
Ins/ins	CC	0.32	0.19	0.18	0.20	0.29	0.30
Ins/del +Del/del	CC	0.13	0.11	0.06	0.12	0.14	0.11
Ins/ins	CT	0.35	0.41	0.37	0.40	0.30	0.36
Ins/del+Del/del	CT	0.12	0.17	0.14	0.12	0.16	0.11
Ins/ins	TT	0.08	0.08	0.13	0.11	0.10	0.11
Ins/del+Del/del	TT	----	0.04	0.05	0.05	0.03	0.01

Usually, TLR2 SNP has indicated like −196 to −174 del. A single band at 286 bp was judged as wild type, and a single 264 bp band was judged as homozygous type, while heterozygous type revealed two bands of 286 bp and 264 bp.

**Table 4:** *TLR2-IL28B* haplotype association with blood donors and HCV-positive patients affected by liver (cirrhosis and hepatocellular carcinoma) or lymphoproliferative diseases (type II cryoglobulinemia and non-Hodgkin's lymphoma) compared with respect to chronic HCV infection (CHC)

	Dominant *(Ins/Ins-C/C)*		Recessive *(del/del-T/T*)		Additive 1 *(hetero vs homoz)*		Additive 2 *(Ins/Ins-C/C vs del/del-T/T)*	
Individual status	*P*-value	OR (95% CI)	*P*-value	OR (95% CI)	*P*-value	OR (95% CI)	*P*-value	OR (95% CI)
*Blood donors*	**0.007**	**2.94 (1.34-6.47)**	0.92	0.85 (0.033-21.27)	**0.030**	**0.44 (0.21-0.92)**	0.718	0.55 (0.021-14.3)
*Hepatic disease*	0.857	1.05 (0.62-1.78)	0.633	0.51 (0.032 −8.18)	0.190	0.75 (0.49-1.15)	0.562	0.47 (0.03-7.17)
*Lymphoproliferative disease*	**0.029**	**1.77 (1.06-2.95)**	0.284	0.17 (0.01-4.30)	0.056	0.66 (0.43-1.01)	0.200	0.12 (0.01-3.05)

Reference category: CHC, chronic HCV-positive patients

Abbreviations: OR, odds ratio; CI, confidential interval.

### Relationship between HCV viral load and TLR2- IL28B haplotype

HCV viral load was assessed in serum samples for 197 patients at diagnosis. A higher HCV viral load was mainly associated with the presence of IL28B C/C rather than that of IL28B T/T genotype (IL28B additive 2, *p* = 0.005, HR 1.68, 95% CI 1.02-2.75, Table 5) and TLR2 ins/del genotype than homozygous (TLR2 additive 1, *p* = 0.026, HR 1.30, 95%CI 0.96-1.73). Multivariate regression analysis indicated that IL28B T/T recessive genotype was independent of HCV genotype, age, patient gender and TLR2 variables and it was associated with a lower HCV viral load (*p* = 0.044, exp 1.52 95% CI 1.01-2.27).

**Table 5:** *TLR2* and *IL28B* genotype and *TLR2-IL28B* haplotype association with HCV viral load

UNIVARIATE ANALYSIS								
	Dominant		Recessive		Additive 1		Additive 2	
	P-value	HR (95% CI)	P-value	HR (95% CI)	P-value	HR (95% CI)	P-value	HR (95% CI)
*TLR2 (ins→del)*	0.564	1.07(0.80-1.44)	0.968	0.95(0.55-1.77)	**0.026**	**1.30(0.96-1.73)**	0.920	1.02(0.57-1.84)
*IL28B rs12979860 (C→T)*	**0.041**	**1.27(0.95-1.68)**	**0.022**	**0.68(0.43-1.07)**	0.691	0.96(0.72-1.26)	**0.005**	**1.68(1.02-2.75)**
*TLR2-IL28B haplotype*	**0.054**	**1.38(1.011-1.87)**	0.823	0.80 (0.09-7.18)	0.44	1.13(0.76-1.68)	0.790	1.29(0.14-12.20)
**MULTIVARIATE ANALYSIS**								
*IL28B rs12979860 (C→T)*	**0.044, exp 1.52 95% CI 1.01-2.27**							

Abbreviations: HR. hazard ratio; CI. confidential interval. Hazard ratio; CI. confidential interval. Hazard ratio; CI. confidential interval. WT: wild type is the most frequent polymorphism found in the general population. For the TLR2 SNP indicated like *ins* and *del* variant; a single band at 286 bp was judged as WT, while heterozygous type revealed two bands of 286 bp and 264 bp (*del* variant). Genotypes of each polymorphism were assessed according to dominant (1 wild-type homozygote; 0 heterozygote and variant homozygote), recessive (0 wild-type homozygote and heterozygote; 1 variant homozygote) and additive genetic models. Additive models comprised additive 1 (1 heterozygotes versus 0 wild-type homozygotes) and additive 2 (1 variant homozygotes versus 0 wild-type homozygotes) models. Dominant indicated homozygous WT alleles, which was for the TLR2 the ins/ins genotype and for the IL28B the C/C genotype. Multivariate stepwise tested for age, sex, HCV genotype, TLR2 and IL28B variables.

### Relationship between liver disease progression and TLR2- IL28B haplotype

**Figure 3:**
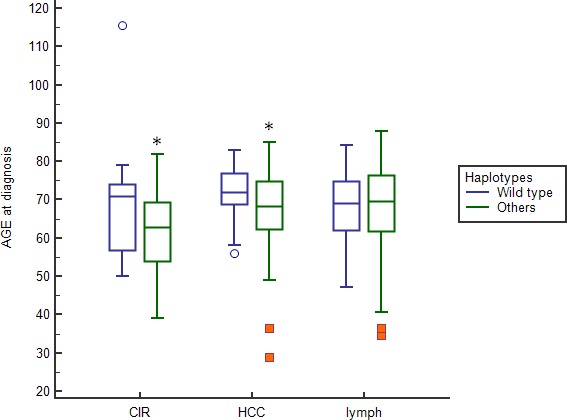
Boxplot describing the relationship of TLR2-IL28B haplotype with the patient disease stratified by the median age at diagnosis Boxes range from the 25th to the 75th percentile with a horizontal black line at the median and vertical lines extending to the 10th and 90th percentiles. The wild type TLR2-IL28B haplotype showed an association with the older patients having a liver disease (patients with HCV-related cirrhosis (CIR)(*)(*p* = 0.038) and patients with HCV-related hepatocellular carcinoma (HCC)(*)(*p* = 0.02). We found no difference in the median age at diagnosis in patients with lymphoproliferative diseases.

Given the potential role of host immunity in the control of HCV-infected tumor cells and the potential of age-related influences on tumor progression, we also looked for a liver disease age-related trend and TLR2-IL28B haplotype. Continuous age variable was analyzed between liver and lymphoproliferative groups with respect to the dominant wild-type TLR2- IL28B haplotype, and a significant association was found with BDs and patients with a lymphoproliferative disease. A significant difference was found in the mean age between patients having a dominant haplotype and a liver disease compared to those having a different haplotype (CIR patients: 68.9 *vs* 62.0 mean age, *p* = 0.038; HCC: 72.4 *vs* 67.7 mean age, *p* = 0.02; Figure 3).

## DISCUSSION

Immunogenetic profile may play an important role in determining the progression of chronic HCV infections to different diseases [1–3, 5, 27]. We confirmed the role of rs12979860 IL28B-C allele-carrier as a protective factor for CHC [2, 17, 21–26]. In addition, we highlighted that the IL28B C/C genotype correlates with lymphoproliferative disease when associated with a TLR2-*ins* allele in homozygosity (Table 4). On the contrary, homozygous TLR2-*del* allele (TLR2-del/del) exhibits a significantly higher threshold for HCC development (Figure 2). Overall, the present results were consistent with an interlocked role between TLR2 and IL28B (IFN-λ3) gene variants in directing HCV-related diseases progression. Indeed, it has been shown that TLR2 was able to activate transcription factors that drive the expression of antiviral genes and different cytokines, such as interferons [15, 16]. Several studies have indicated a relation among rs12979860 IL28B gene, spontaneous clearance of HCV and therapeutic outcomes of genotype 1 HCV-positive patients treated with IFN-based therapies [2, 21–26]. In 2013 a new interferon, the IFN-λ4, was identified. The rs368234815 polymorphism originating the IFN-λ4 by creation of a new open reading frame [19, 28] was found in strong linkage disequilibrium with the rs12979860 IL28B so much it has been described as the functional variant of the rs12979860 IL28B [19, 29]. Thus, spontaneous and treatment-induced clearance of HCV resulted equally dependent on rs12979860 or rs368234815 polymorphisms and combined assessment of these gene polymorphisms did not increase the predictive value of IL28B polymorphisms in HCV-positive patients [19, 29]. More recently, it was found that Y93H, a variant present in the NS5A region of HCV, was significantly associated with the beneficial rs12979860 polymorphism confirming the functional role of IFN-λ4 SNP in HCV-infection [30]. The haplotype data presented herein (Table 4) provided evidence supporting a role for IL28B in relation with TLR2 polymorphism to predict progression to different HCV-related diseases. Recent functional studies have demonstrated that IL28B T/T genotype was associated with a higher production of IFN-λ3 and higher ISGs gene expression in HCV-infected liver tissue, and that this higher baseline level of ISGs expression may contribute to an interferon-refractory state in the liver [31]. Thus, it has been hypothesized that the high baseline ISG levels found in IL28B T/T carriers can lead to a poor response to INF-based therapy due to an exhaustion of the interferon-response pathway [32]. In keeping with this model, a satisfactory treatment response could be associated with a IL28 C/C genotype, a lower levels of ISGs expression and thus a higher levels of circulating HCV RNA [33]. Our findings about higher viral load in patients having IL28B C/C genotype may support this hypothesis. A higher viral load was associated with HCV genotype 1, but difference among genotypes did not reach a statistical significance in our series. The observed broader frequency of IL28-T allele in patients with hepatic rather than lymphoproliferative disorders can be explained by the fact that the IL28-R/IL-10R receptor complex was expressed only in restricted cell types, like hepatocytes, epithelial cells and plasmacytoid dendritic cells [34]. Considering that HCV was primarily hepatotropic, it is reasonable to assume that IL28B-T allele could likely contribute to an IFN-refractory state mostly in the liver. In this regard, it has been observed that the expression levels of ISGs were differentially regulated in the liver and peripheral blood [35, 36]. In addition, higher level of HCV infection in hepatocytes induced higher level of endoplasmatic reticulum stress and caused HCC carcinogenesis [37]. In this scenario, TLR2-*del* variant, which was related to a diminished expression of TLR2 level, and secretion of immunosuppressive cytokines, increased the hepatic inflammatory microenvironment and the risk of HCC development [8, 13, 38]. Consistently, TLR2 knock-down (TLR2*^−/−^*) mice showed enhanced inflammation, hepatic lesions and HCC progression after carcinogenic diethylnitrosamine exposure [9, 39]. Moreover, the TLR2 del/del condition resulted more frequently detected in our HCC patients than in other HCV-related diseases (Figure 2). Data were in agreement with those observed in the HCV German population with an increased risk of HCC associated with the TLR2-*del* variant [13]. On the other hand, our multivariate analysis showed that IL28B C/C genotype was associated with a higher viral load (Table 5) and that the dominant wild-type haplotype (TLR2 *ins/ins*-IL28B C/C) correlated with lymphoproliferative diseases but also with BDs (Table 4). Likewise, previous studies have shown that HCV-core protein triggering TLR2 leaded to an increased in B-cell proliferation *in vitro* [18]. Thus, considering that HCV is also a lymphotropic virus, our haplotype analysis pointed out that in the presence of a HCV chronic persistence, the TLR2 *ins/ins*-IL28B-C/C arrangement could increase the susceptibility to lymphoproliferative complications, including mixed cryoglobulinemia and B-cell NHL (*p* = 0.029, OR 1.77, Table 4). In addition, the TLR2 *ins/ins*-IL28B-C/C haplotype not only showed a potential protective effect against HCV infection but also an association with older patients having a liver diseases (CIR and HCC, Figure 3), thus, suggesting that the protective effect associated with this haplotype decreased with age.

In conclusion, our study emphasizes that both TLR2 and IL28B polymorphisms may have a role in directing HCV-progression towards hepatic or lymphoproliferative diseases. Thus, the analysis of our HCV-positive population stratified by the two immunological variables indicated a potential protective effect of the IL28B-C allele in homozygosity (IL28B-C/C) towards chronic infection and liver diseases, and that the simultaneous presence of at least one TLR2-*del* allele abolished this effect. In accordance with this result, the TLR2-*del/del* state appeared closely linked with HCC condition. Conversely, the dominant wild-type haplotype (TLR2 *ins/ins*-IL28B C/C) associated with a better spontaneous resolution of the infection was also found more frequently in both BDs as well as in HCV-related lymphoproliferative disorders compared to CHC, suggesting that, in patients with a lymphoproliferative disease, additional factors may be involved that obstacle the elimination of the virus. Therefore, our findings shed new light on the mechanisms underlying the persistence of HCV infection and HCV-related disease progression. A deeper understanding of these processes may lead to development of strategies based on the combination of conventional therapies with immune-mediated strategies for the treatment of HCV-related diseases.

## MATERIALS AND METHODS

### Study samples

We considered 862 patients with chronic HCV-infection and 77 healthy blood donors. The ethnic background of patients was caucasic, without HBV or HIV infection. Among HCV-positive patients, 230 had a chronic HCV infection (CHC *n* = 230), 123 patients had cirrhosis (CIR *n* = 123), 175 patients had hepatocellular carcinoma (HCC), and 334 patients had a lymphoproliferative disease including: 205 a cryoglobulinemic syndrome (MCS) defined according to previously described criteria [3] and 129 an overt B-cell non-Hodgkin's lymphoma (NHL). Demographic and clinical characteristics of each groups were reported on Table 1. Patients had been recruited from: the MASVE Center, University of Florence, Azienda Ospedaliera Santa Maria degli Angeli, Pordenone, National Cancer Institute “Fondazione Pascale”, Naples, Departement of Biomedical Sciences and Human Oncology, University of Bari Medical School, Policlinico Universitario di Udine and Centro di Riferimento oncologico di Aviano, Italy. The diagnosis of chronic HCV infection was based on anti-HCV, elevated ALT serum levels and HCV RNA positivity of at least 6 months duration. An immunoassay test (III-generation EIA) against HCV-core and HCV-non structural antigens were used. HCV genotype was determined by a commercial, certified, diagnostic test (Versant HCV Genotype 2.0, Siemens Healthcare Diagnostics, Deerfield, IL). Quantitative determination of HCV loads (RNA UI/mL) was done by branched DNA technology (Chiron, Emeryville, CA). The diagnosis of HCC was based on the standard criteria listed in the European Association for the Study of the Liver (EASL) that incorporate both invasive and noninvasive measures. Noninvasive criteria include two imaging techniques, both demonstrating a focal lesion > 2 cm in diameter with features of arterial hypervascularization. Detection and immunochemical characterization of cryoglobulins were performed according to consensus protocol proposed by A.L.CRI (Associazione Italiana per la Lotta alle Crioglobulinemie). NHL in the course of HCV infection has been histopathologically confirmed based on WHO classification [3]. The study was conformed to the ethical guidelines of the Helsinki Declaration and all subjects provided informed consent. Study was approved by independent local and independent ethics committees since this was a multicenter study.

### Genetic polymorphisms of TLR2 and IL28B

Genomic DNA was extracted from whole blood using the EZ1 Qiagen blood kit and protocols recommended by the manufacturer (Qiagen Inc., Valencia, CA). Determination of TLR2 −196 to −174 ins/del polymorphism was performed by Polymerase Chain Reaction (PCR) using the 5′-ctcggaggcagcgagaaa-3′ and 5′-ctgggccgtgcaaagaag-3′ primers (10 pmol) in a reaction volume of 25 μl including 200 ng dNTPs and 0.5 U of GoTaq DNA Polymerase (Promega Corporation, Madison, WI). Cycling conditions were: initial denaturation at 95°C for 5 min, followed by 35 cycles at 95°C for 30 sec, at 60°C for 40 sec and at 72°C for 40 sec and then 72°C for 7 min. Fragments of different length (264 bp and 286 bp), depending on the presence or absence of the deletion mutation were visualized by electrophoresis on a 3.5% agarose gel staining with ethidium bromide. IL28B genotyping was performed using a specific custom TaqMan SNP-genotyping Assay (SNP rs12979860; Applied Biosystem, Foster City, CA, USA) based on allele-specific dual-labelled probes on a 7900HT Fast Real-Time PCR system (Applied Biosystem, Foster City, CA, USA). Amplicon sequencing was used to validate the genotyping techniques.

### Statistical analysis

Specific tests including Fisher's exact test and one or two-way analysis of variance were used to compare the patient groups regarding allele and genotype frequencies of TLR2 and IL28B polymorphisms, by direct counting of the positive individuals for a specific allele/genotype polymorphism. Multivariate logistic regression analysis was performed with diagnosis as a dependent variable and independent variables, including age, gender (0 female; 1 male), and each genotype were also considered. P-value, odds ratio and 95% confidence interval were calculated. Genotypes of each polymorphism were assessed according to dominant (0 wild-type homozygote; 1 heterozygote and variant homozygote), recessive (0 wild-type homozygote and heterozygote; 1 variant homozygote) and additive genetic models. Additive models comprised additive 1 (heterozygotes *versus* wild-type homozygotes) and additive 2 (variant homozygotes *versus* wild-type homozygotes) models, which were analyzed simultaneously with a single statistical model. Statistical analyses were performed using GraphPad Prism v6. *P* value < 0.05 was considered significant.
